# Identification of 34 Novel Proinflammatory Proteins in a Genome-Wide Macrophage Functional Screen

**DOI:** 10.1371/journal.pone.0042388

**Published:** 2012-07-31

**Authors:** David H. Wyllie, Karen C. Søgaard, Karen Holland, Xu Yaobo, Migena Bregu, Adrian V. S. Hill, Endre Kiss-Toth

**Affiliations:** 1 Jenner Institute, Old Road Campus Research Building, Oxford University, Oxford, United Kingdom; 2 Department of Cardiovascular Science, University of Sheffield, Sheffield, United Kingdom; 3 Institute of Cellular Medicine, Newcastle University, Newcastle, United Kingdom; Centro Cardiologico Monzino IRCCS, Italy

## Abstract

Signal transduction pathways activated by Toll-like Receptors and the IL-1 family of cytokines are fundamental to mounting an innate immune response and thus to clearing pathogens and promoting wound healing. Whilst mechanistic understanding of the regulation of innate signalling pathways has advanced considerably in recent years, there are still a number of critical controllers to be discovered. In order to characterise novel regulators of macrophage inflammation, we have carried out an extensive, cDNA-based forward genetic screen and identified 34 novel activators, based on their ability to induce the expression of *cxcl2*. Many are physiologically expressed in macrophages, although the majority of genes uncovered in our screen have not previously been linked to innate immunity. We show that expression of particular activators has profound but distinct impacts on LPS-induced inflammatory gene expression, including switch-type, amplifier and sensitiser behaviours. Furthermore, the novel genes identified here interact with the canonical inflammatory signalling network via specific mechanisms, as demonstrated by the use of dominant negative forms of IL1/TLR signalling mediators.

## Introduction

It is widely believed that a significant fraction of mammalian genes is involved in mediating resistance to pathogens [1]. Many of these form part of innate signalling systems, a comprehensive understanding of which remains elusive despite intense study, partly because many components are still to be characterised. Cataloguing the components of these signalling processes and their interactions is a key goal of systems biology research [2]. Moreover, many of the presently unrecognised components may become targets for future drug development in the context of inflammatory disease.

Historically, several strategies have been employed to identify such components, ranging from expression cloning of cytokines and their receptors [3], genetic studies using mutant cells with impaired ability to respond to inflammatory stimuli [4], protein-protein interaction screens [5], homology-based identification [6], and computational analysis of emerging genomic datasets [7].

These efforts have led to the development of the current model of inflammatory signalling, whereby members of the IL-1R/TLR family activate signalling via a group of adaptor proteins, MyD88, Mal/TIRAP, TRIF and TRAM [8]. Upon receptor-ligand interactions, further proteins are recruited to these multi-protein complexes, including members of the IRAK kinase family [9]. This then leads to activation of TRAF6 ubiquitin ligase, which interacts with TAK1 and TAB proteins and thus activates downstream signal processing pathways, including the IκB/NFκB complex and MAPK networks [10], [11]. In addition to these, distinct TLR receptors are able to induce cellular responses via activating members of the IRF transcription factor family, namely IRF3 and IRF7, in order to drive expression of type I interferons (ifn) [8]. For this, the serine/threonine kinase TBK1 is essential for phosphorylation and hence activation of IRF3 and IRF7 [12]. TBK1 is also critical for signalling by non-TLR intracellular pathogen signalling systems, including DNA sensing systems [13]–[15].

It is however clear that many components of innate signalling pathways remain to be identified, most notably those which control specificity and the amplitude of action or the previously unrecognised genes interacting with the canonical signalling network via novel mechanisms [1]. In recent years, we have developed a screening platform that takes advantage of transcriptional reporters inducible by inflammatory stimuli. We have demonstrated the utility of this system by identifying previously unknown proteins, the overexpression of which regulates the activity of these reporters. Examples of these include the family of tribbles proteins, the biology of which we have characterised [16]–[18], miR155/BIC [19], [20], and Jmj/JARID [21], [22]. This platform has also been adopted by others and has been used successfully to identify a large number of novel signalling regulators, with the aim of developing these as drug targets [23]. Similar approaches led to the recent identification of ERIS/STING and RIG-1 as novel regulators of interferon activation [24], [25].

Here we report the screening of a cDNA collection of 30,000 fully characterised murine expression clones, representing over 12,000 unique genes in Raw 264.7 macrophage-like cells, and the identification of 34 mostly uncharacterised proteins which activate the expression of a canonical target of pro-inflammatory signalling systems, the *cxcl2* (MIP-2) chemokine.

## Results

### Generation of a fully characterised, genome-wide mouse cDNA collection

#### Library construction and sequencing

Three cDNA libraries were created from mouse embryo, brain and spleen. 50,000 clones were picked from each of the three libraries, and plasmid DNA from each was sequenced from the 5′ end (see File S1). Later in the process, selected clones were also sequenced from the 3′ end (see File S2).

A total of 140,000 5′ sequence reads were obtained and analysed: 118,490 sequences passed QC using Phred; following vector clipping, >50 bp high quality sequence remained in 98,918 sequences which were mapped to the mouse genome using Blat. Of these sequences, blat hits were found for 93,009 sequences (94%), which were clustered into 13,909 clusters. The reasons for the absence of hits in the other 6% are probably that they represent highly repetitive sequence. The alignment revealed that sequenced clones corresponded to 7,041 genes on the sense strand and matching at least 2,900 genes on the antisense strand; including 758 sequences matching genes on both the sense and antisense strands. 8% of the 98,918 sequences did not match a known gene or gene model. The three libraries were re-arrayed into one non-redundant library and supplemented with 10,000 cDNA clones in the same expression vector from the IRAV part of the mammalian Gene Collection (Source Biosciences, File S4). The resulting non-redundant library contains 13,239 unique reference sequences, ∼45% of canonical mouse genes (http://www.informatics.jax.org/genes.shtml) and the total number of clones is approx. 30,000 (see File S5). Consequently, a number of genes are represented by several splice variants and/or non-protein coding mRNA species with putative regulatory function. In order to simplify further referencing of our results obtained by testing this cDNA collection, we named it the Mouse Transcriptome Collection (MTC).

### Identification of 34 activators of *cxcl2* expression in Raw 264.7 cells

**Figure 1:**
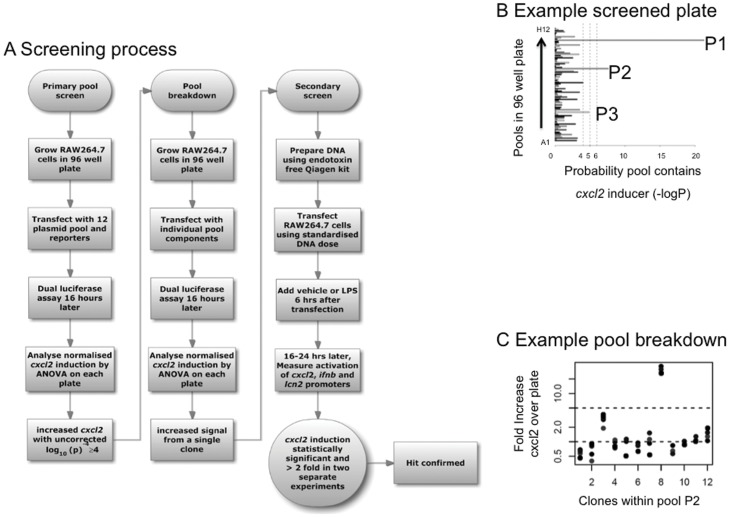
Flow chart of the functional screen. **A**) shows the workflow of the screen. **B**) shows an example of the analysis performed on a plate comprising 96 pools of 12. The hypothesis that individuals pools differ from the plate mean is tested using a linear model. Three pools (P1, P2, P3) give signal over the −logp>4 cut off used. Breakdown of pool P2 is shown in **C**) one constituent clone (#8) gives high-level induction. Dots represent results of four independent replicates, performed for each clone.

**Figure 2:**
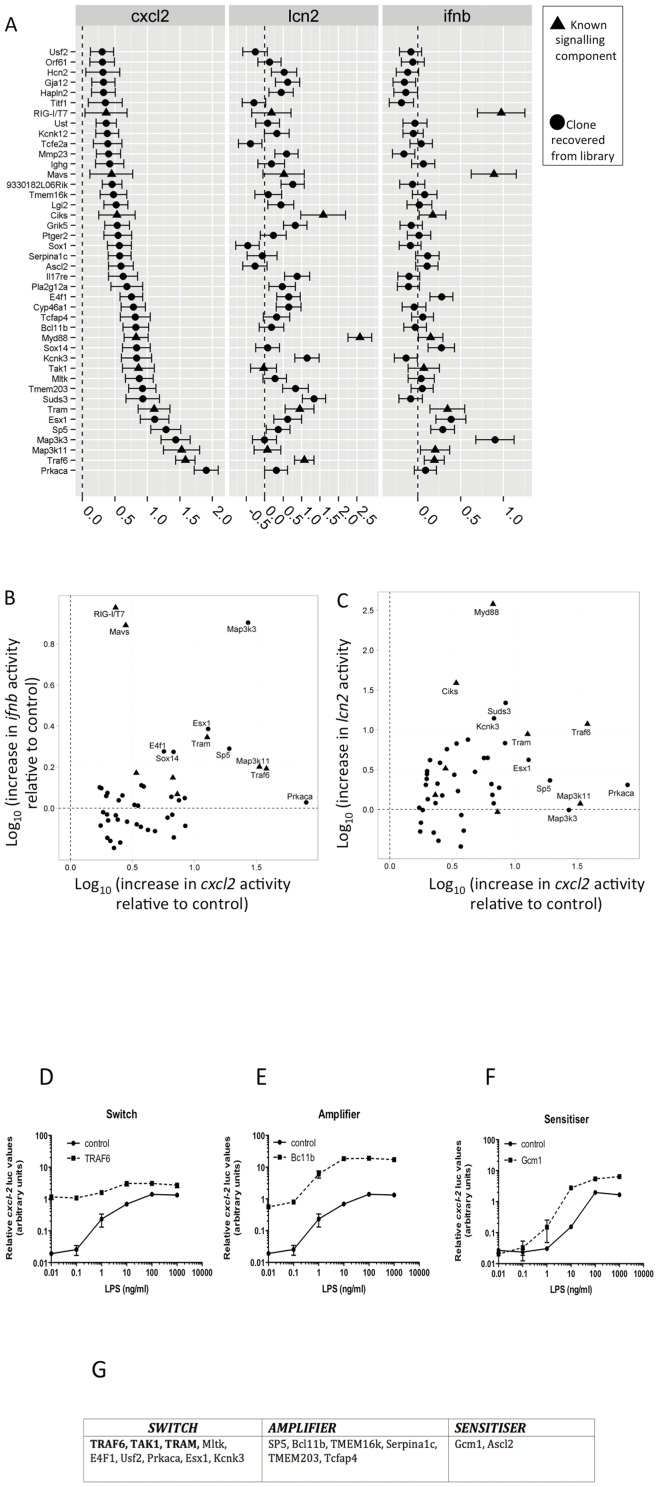
Activation of proinflammatory promoters by library hits and their impact on LPS induced *cxcl2* activation in Raw 264.7 cells. Induction of three proinflammatory promoters (*ifnb*, *cxcl2* and *Lcn2*) by plasmids recovered from the library screen and positive controls are shown. Results are derived from 2 (for *ifnb* and *lcn2*) and between 3 and 13 independent experiments (for *cxcl2*), all performed in triplicate in each experiment. **A**) Results are expressed as log fold increase of normalised promoter activity over background and 95% confidence intervals. **B**) and **C**) show bivariate plots illustrating activity of the various molecules on the *cxcl2*, *lcn2* and *ifnb* reporters. Effect estimates for molecules derived from the screen are marked as circles, and known components of signalling system as triangles. **D–F**) Raw 264.7 cells were transfected with the *cxcl2*-pLuc and EF1-rLuc reporters, as described in the methods, as well as with 60 ng/well expression plasmid, encoding known (TRAF6) (**D**) or novel proinflammatory molecules (**E** and **F**). The impact of over-expressed proinflammatory mediators on LPS induced *cxcl2* expression was tested. The activity profile of the cDNAs tested were classified according to three distinct patterns, as exemplified in D–F and shown in **G**). Genes highlighted in bold encode known components of inflammatory signal transduction (controls).

For high throughput screening of MTC (outlined in figure 1A), plasmid preparation was performed for each clone and aliquots of these were pooled into pools of 12 which were subsequently transfected into Raw 264.7 cells, in triplicate (Figure 1B). Analysis of variance (ANOVA) was used to identify pools that were able to significantly enhance *cxcl2* reporter activity above the background. Clones from positive pools were tested individually and the cDNA clone activating the *cxcl2* reporter was identified (Figure 1C). 61 positive clones identified in the initial screen (Table 1) were subsequently re-tested in further, independent transfections using DNA purified using methods designed to minimise endotoxin contamination (Figure 2A and highlighted in Table 1). 34 genes induced *cxcl2* activation reproducibly and statistically significantly by at least two-fold, which is close to the detection limit in this system.

**Table 1:** Genes activating *cxcl2*-pLuc expression in Raw 264.7 cells.

Murine gene ID	Human gene ID	Gene name	Predicted localisation	Expression in macrophages	Clone length
**210741**	**56660**	**Kcnk12**	Plasma membrane (P)		N del
**16527**	**3777**	**Kcnk3**	Plasma membrane (E)		C del
18830	5360	Pltp	Secreted (E)	+++	
**338362**	**10090**	**Ust**	Golgi, type II transmembrane protein (P)	++	
**73940**	**60484**	**Hapln2**	Secreted		C del
23934	4062	Ly6h	Plasma membrane (E)		
**118454**	**57165**	**Gjc2/Gja12**	**Plasma membrane**	**+**	
72114	84327	Zbed3	Intracellular	+	
**20669**	**8403**	**Sox14**	Nucleus (P)	+	
16319	3619	Incenp	Nucleus (P)	++	
72961	57030	Slc17a7	Plasma membrane		
16478	3727	Jund1	Nucleus (E)	+	
234023	55082	Arglu1	Nucleus (P)		
**14809**	**2901**	**Grik5**	Cytoplasmic vesicles (E)		N del
631309	57719	TMEM16H/ANO8	Plasma membrane (P)	+	N del
**15166**	**610**	**Hcn2**	Plasma membrane (P)	+	N del
**21869**	**7080**	**Titf1/NKX2-1**	Nuclear (E)	**+**	N del
**216157**	**91304**	**ORF61/membralin**	Plasma membrane (P)	++	

Murine gene ID	Human gene ID	Gene name	Predicted localisation	Expression in macrophages	Clone length
353156	51162	Egfl7	Secreted (P)		
**13116**	**10858**	**Cyp46a1**	**Secreted (P)**		
**71954**	**64426**	**Suds3**	Nuclear (E)	+	
**231014**	**222223**	**9330182L06Rik/KIAA1324L**	Unknown	+	
21885	7088	Tle1	Nucleus (P)		
12153	649	Bmp1	Secreted (E)		C del
631901	6658	LOC631901	Unknown		
**58208**	**64919**	**Bcl11b**	Nucleus (P)		N del
**26561**	**8510**	**Mmp23**	Extracellular (P)		
**64406**	**389058**	**Sp5**	Nuclear (E)		
67674	51504	Trmt112	Unknown	+	
**13984**	**80712**	**Esx1**	Nucleus (P)	+	
**102566**	**55129**	**Tmem16k/ANO10**	Plasma membrane (P)	++	
**22282**	**7392**	**Usf2**	Nucleus (E)	+++	N del
74123	116113	Foxp4	Nucleus (P)	+	
**13560**	**1877**	**E4f1**	Nucleus (E)	+	
53381	10549	Prdx4	Cytoplasm (E)	++	
**18747**	**5566**	**Prkaca**	Cytoplasm (P)	++	
**19217**	**5732**	**Ptger2**	Plasma membrane (P)	+++	
14161	2243	Fga	Secreted (E)		
**21423**	**6929**	**Tcfe2a/TCF3**	Nucleus (E)	+	
23872	2114	Ets2	Nucleus (E)	+++	

Murine gene ID	Human gene ID	Gene name	Predicted localisation	Expression in macrophages	Clone length
75965	253832	Zdhhc20	Plasma membrane (P)		
**20702**	**5265**	**Serpina1c**	Secreted (E)		
**17173**	**430**	**Ascl2**	Nucleus (E)	+	
**66350**	**81579**	**Pla2g12a**	ER (E)	++	
**380794**		**Ighg**	Secreted (P)		
**227615**	**94107**	**Tmem203**	Multipass transmembrane (P)	++	
**26406**	**4215**	**Map3k3**	Cytoplasm (E)	+++	
13649	1956	Egfr	Plasmamembrane (E)		
240913	9507	Adamts4	Secreted (E)		
**65964**	**51776**	**B230120H23Rik/Mltk/ZAK**	Cytoplasm, nucleus (E)		
67216	129642	Mboat2	Multipass membrane, ER (P)		
76905	116844	Lrg1	Nucleus (E)		
66950	55248	2310028N02Rik/TMEM206	Cytoplasm, nucleus (P)	+++	
237553	29953	Trhde	Membrane (P)		
**83383**	**7023**	**Tcfap4/TFAP4**	Nucleus (E)	+	
21750	7014	Terf2	Nucleus (E)		
78755	159090	Fam122b	Nucleus (P)		
14531	8521	Gcm1	Nucleus (P)	+	
97114	8352	Hist2h3c2	Nucleus (E)	++	
**246316**	**55203**	**Lgi2**	Secreted (P)		
102954	170685	Nudt10	Nucleus (P)	+	

Murine gene ID	Human gene ID	Gene name	Predicted localisation	Expression in macrophages	Clone length
57890	132014	**IL17re**	Membrane (E)		
20664	6656	**Sox1**	Nucleus (E)		

Genes encoding the mouse clones uncovered in the functional screen were identified in GenBank. Putative localisation of the various proteins have been assessed using LOCATE (http://locate.imb.uq.edu.au/). Predicted localisation is indicated by (P), and experimentally verified cellular localisation is indicated by (E). Endogenous mouse macrophage expression of the genes identified here has been characterised using BioGPS (http://biogps.gnf.org/). The relative abundance of macrophage expression, compared to other cell types have been indicated (“+++” is for high expression levels, “+” is for modest expression). Genes that induced *cxcl2* expression in the absence of residual Pathogen-associated molecular patterns (PAMP) contamination are highlighted in bold. Genes in bold induced the *cxcl2* reporter >2 fold under strictly endotoxin-free conditions.

C del denotes a C-terminal deletion in the library clone relative to the reference protein; N del denotes a N-terminal deletion in the library clone relative to the reference protein.

Initial computational analysis revealed that the hits include predicted secreted, transmembrane, intracellular and nuclear proteins (Table 1). As shown in Table 2, genes with common biological action (ion channels, transcription factors) and components of common biological pathways (matrix modifiers, phospholipid homeostasis) have been identified by the screen, suggesting an important regulatory function for genes with such activity in controlling macrophage inflammation. Further, using publically available expression profile data at the BioGPS portal, we showed that the majority of the proteins identified in the screen are endogenously expressed in macrophages (Table 1). Interestingly, analysis of transcripts for the hits revealed that 10 of these contained cDNAs encoding only partial open reading frames (Table 1), leading to the expression of truncated proteins. This observation is in line with our previous findings, demonstrating that “full-lengthness” is not a necessary requirement for the detection of molecules with inflammation modulatory action [26].

**Table 2:** Functionally linked groups of proteins uncovered in the screen.

Ion channels	Transcriptional regulators	Modifiers of Extracellular Matrix	Signalling molecules	Phospholipid homeostasis
Kcnk12, Kcnk3Gjc2, Slc17a7Hcn2,ANO8, ANO10	Sox14, IncenpJunD1, Titf1Suds3, Tle1Bcl11b, Sp5Esx1, Usf2Foxp4, E4F1Ets2, Ascl2Tcfap4, Terf2,Sox1	Ust, HalpnMmp23, ADAMTS4	Prkaca, MAP3K3ZAK, Lrg1	PltpPla2g12aMboat2

Groups of proteins sharing similar biological function and modifiers of common processes identified as *cxcl2* activators in the functional screen.

### Characterisation of the biological activity and specificity of selected hits and their connections with inflammatory pathways

Following the isolation of the 34 genes described above, further transient transfection experiments were performed to characterise their bioactivity using two additional transcriptional reporters, driven by the promoters of the lipocalin 2 (*lcn2*) and the interferon beta (*ifnb*) genes. These promoters were chosen by analysing microarrays of stimulated Raw 264.7 cells and identifying genes that were activated by proinflammatory stimuli but with distinct kinetics. Details of our analysis and target selection for these assays have previously been described [27]. We included 8, well characterised pro-inflammatory signalling molecules (MAVS, Traf3IP2/Ciks/Act1, Myd88, Tak1, Tram, Map3k11, Traf6, and RIGI/T7) in these assays. Most of the 34 genes found were weak *ifnb* inducers in Raw 264.7 cells (Figure 2A and B), with the exception of MAP3K3, which induced levels of *ifnb* comparable with the canonical *ifnb* inducers RIG-I (stimulated by T7-produced transcripts) or MAVS. In contrast, activation of the *lcn2* promoter correlated well with that of the *cxcl2* promoter, although genes that preferentially induced one, but not the other were also observed, including MyD88, Traf3IP2 and Prkaca (Figure 2A and C).

A range of modulators of signalling induced by TLR and proinflammatory cytokine receptors have been uncovered during the past decade and a number of variants of these genes have recently been identified as risk factors for inflammatory disease [28], [29]. Therefore, we have investigated how elevated levels of 13 transcripts identified in the Raw 264.7 cell screen above would affect LPS induced *cxcl2* transcription. The genes analysed here were selected to include molecules with a range of predicted intracellular localisations (transmembrane proteins, intracellular signalling molecules and transcription factors). Three well-characterised mediators of inflammatory signalling were used as positive controls (TRAF6, TAK1 and TRAM). Raw 264.7 cells transfected with empty vector or a proinflammatory mediator were stimulated by increasing doses of LPS for 6 hrs and the activity of the *cxcl2* promoter was measured (Figure 2D–F). All the known proinflammatory signalling molecules tested, as well as 43% (6 of 14) of the novel proteins identified in our screen induced maximal induction of *cxcl2* in the absence of LPS (Fig. 2D and 2G, “switch” type response). In contrast, the remaining molecules exhibited a different relationship with the LPS-induced reporter responses: overexpression of 43% (6 of 14) of genes tested amplified LPS-induced reporter activation, increasing the maximal response (Fig. 2E and G, “amplifier” response); the remaining 2 of 14 led to a reproducible and highly statistically significant shift in the EC_50_ of the LPS induced dose-response, suggesting that these proteins are able to sensitise the cells to LPS (Fig. 2F and G, “sensitiser” response).

Thus, genes with diverse modes of action were identified in the Raw 264.7 cell screen. However, the majority of these genes and their protein products are not known to be involved in inflammatory signal processing, and for a number of them there is no published literature at all. Therefore, we characterised the molecular mechanisms by which selected novel mediators interact with the canonical inflammatory signalling machinery. Induction of the cxcl2 reporter was monitored on co-transfection of combinations of one dominant negative (DN), inhibitory molecule, and one library hit. LPS was used as a positive control. Six novel pro-inflammatory genes (a poorly characterised protein kinase Mltk/ZAK; ion channels Kcnk3 and Kcnk12; a sulfotransferase UST; a regulator of histone acetylation Suds3, and the transcription factor Bcl11b) were tested in these experiments. The genes were chosen on the basis of novelty to macrophage inflammation, their diverse putative mode of action as well as their potency in activating *cxcl2* expression in our reporter assay.

**Figure 3:**
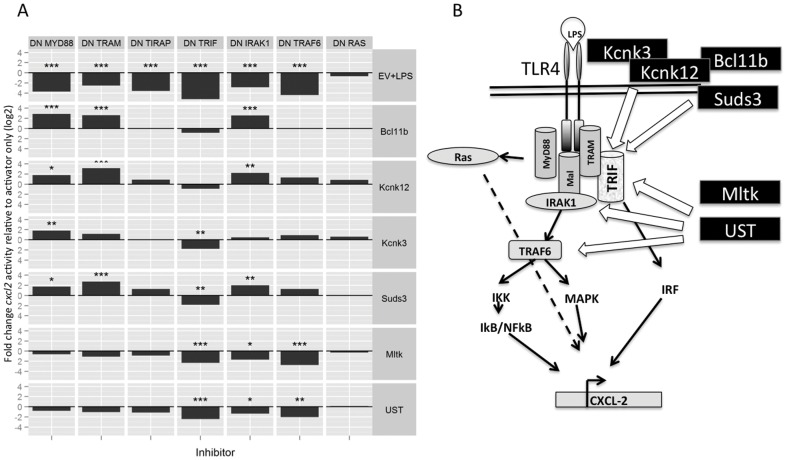
Screen hits interact with the canonical proinflammatory signalling network via distinct molecular mechanisms. **A**) Raw 264.7 cells were transfected with the *cxcl2*-pLuc and EF1-rLuc reporters, as described in the methods, as well as with 30 ng/well expression plasmid, encoding for dominant negative (DN) mutants of known proinflammatory molecules (MyD88, TRAM, TIRAP, TRIF, IRAK1, TRAF6 and Ras, respectively). LPS (100 ng/ml) was used as a positive control (6 hrs stimulation) to test for the inhibitory activity of the DN constructs used. Data are derived from three independent experiments, as described in Methods. *p<0.05 , **p<0.01, ***p<0.001 **B**) summarises the interactions observed in **A**.

**Figure 4:**
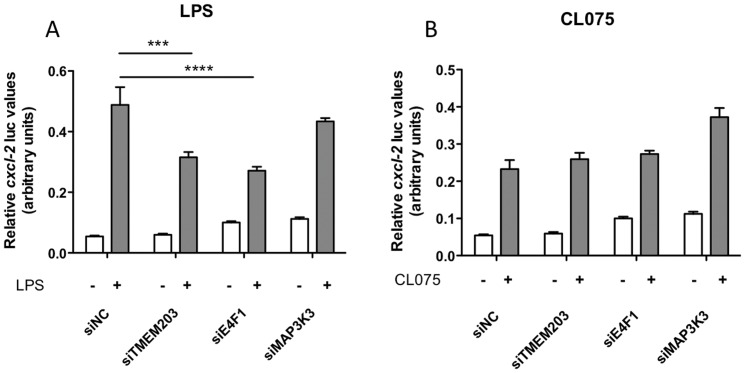
Selected screen hits are required for TLR4 but not for TLR7/8 induced cxcl2 induction. Raw 264.7 cells were transfected with the *cxcl2*-pLuc and EF1-rLuc reporters, as described in the methods, as well as with 10 pmol siRNA (ON-TARGET *plus* SMART pool, Dharmacon) aginst selected hits that are endogenously expressed in these cells. The impact of siRNA knockdown on **A**) LPS (20 ng/ml) or **B**) CL075 (2 µg/ml) induced *cxcl2* reporter activation was measured after 6 hrs stimulation. White bars: vehicle, grey bars: agonist added. Data was analysed by two-way ANOVA modelling effect of agonist (LPS or CL075) and siRNA (four categories). Significance was determined from contrast estimates from the model (***p<0.001, ****p<0.0001). A representative experiment of three, with similar results, is shown.

As shown in Figure 3, the DN mutant proteins, which block the TLR induced inflammatory signal transduction pathway at specific points, included TIRAP, MyD88, TRAM, TRIF, TRAF6, IRAK1 and Ras, respectively. As expected, LPS induced *cxcl2* expression was inhibited by the various DN expression constructs previously reported to affect TLR-mediated inflammatory signals [30]–[35] (Fig. 3A), with the exception of Ras, the action of which is dependent on extracellular matrix-cell interactions [36]. The novel genes, however, generated distinct signals in this assay. For example, the ability of Mltk to drive *cxcl2* induction was significantly inhibited by DN-IRAK1, -TRIF and -TRAF6, indicating that Mltk is likely to act at the level of the receptor complex, at the membrane (Figure 3A). A similar pattern of inhibition was observed when UST induced *cxcl2* expression was inhibited by the DN constructs. In contrast, Kcnk3 -driven *cxcl2* expression was only inhibited by DN-TRIF. Whilst these results suggest that groups of novel signalling intermediates identified in the screen may act via distinct but specific mechanisms, detailed understanding of the molecular basis of these functional interactions will require further investigation. A separate characterisation of the likely physiological role of three hits (TMEM203, E4F1 and MAP3K3) that are endogenously expressed in Raw 264.7 cells on TLR agonist induced activation of *cxcl2* expression is shown in Fig. 4. siRNA mediated knockdown of TMEM203 and E4F1 expression led to impaired *cxcl2* expression in response to LPS, whereas knockdown of MAP3K3 expression had no significant impact in this system (Fig. 4A). In contrast, induction of *cxcl2* expression by the TLR7/8 agonist CL075 was not impaired by knockdown of any of the genes tested (Fig. 4B), suggesting that the proinflammatory mediators isolated in this screen may have a distinct function in controlling signalling pathways that are induced by specific TLRs.

## Discussion

In the study described here, we have undertaken a cellular screen for novel proinflammatory genes, detecting inducers of the chemokine gene, *cxcl2*/in the macrophage-like cell line Raw 264.7. The screen was extensive, and relied on both a low redundancy cDNA library and our previously developed screening platform [26], [27], [37]–[39].

We discovered at least 34 genes which induced the *cxcl2* reporter, most of which were not previously known to be involved in proinflammatory signalling. As discussed below, independent biochemical experiments support a physiological role for many of these genes. The high yield of previously unknown signalling molecules from the screen may be related to the cell line used for screening (Raw 264.7), which is widely recognised to retain many properties of primary macrophages. As such, its phenotype differs markedly from the readily manipulated epithelial cell lines (HeLa, HEK293, etc.) which have been used in previous transcriptome screens for inflammatory molecules. We note that *in silico* analysis of the cell-type specific expression profile of hits from the screen showed that the majority of the genes recovered in this exercise are endogenously expressed in macrophages.

Among the 34 genes found, there are differences in their ability to activate other, inflammation sensitive reporters, and they also have a differential influence on LPS induced *cxcl2* activation (see Fig. 2). Some exhibit “switch on” like behaviour: on molecule overexpression, the cell produces chemokines with limited impact of additional endogenous stimuli. Others exhibit “amplifier” behaviour, shifting the LPS dose-response upwards. Genes in the third category, “sensitisers” shift the LPS induced maximal *cxcl2* activity leftward. We suggest that these latter two groups of genes may be of significant physiological importance in the development and maintenance of inflammatory disease. Additional support for a physiological role for some of the genes found in this screen in inflammatory signalling in macrophages comes from siRNA studies in which 2 out of the three novel molecules tested impaired LPS-induced signalling.

Genetic variants leading to an increase in the expression of such genes would be postulated to lead to a hyper-responsive, inflammatory phenotype, thus contributing to the susceptibility to diseases with a significant inflammatory component. We have recently reported the existence of such a phenotype in periprosthetic osteolysis, a condition that is driven by inflammatory macrophages [40]. Whilst the molecular basis of this hyper-inflammatory phenotype in osteolytic individuals is unclear, genetic variants of genes reported in here may significantly contribute to the development of this condition.

In addition to distinct influences on LPS mediated *cxcl2* activation, experiments outlined in Figure 3 investigated the molecular pathways by which a subset of our hits and the canonical TLR signalling pathway interact. Although a wide range of dominant negative molecules severely attenuated LPS signalling, inhibition profiles for hits differed markedly. This emphasises that, as reported by recent proteomic studies uncovering many novel proteins mediating TNF signalling [5], many aspects of macrophage inflammation remain poorly understood.

Whilst a number of genes identified in this study have the characteristics of “canonical” signalling mediators and their effectors, including MAP kinases and several transcription factors, it is clear that many of the novel hits may highlight the importance of additional regulatory mechanisms of inflammatory gene expression. For instance, two members of the tandem pore K^+^ channel family (Kcnk3 and Kcnk12) have been identified in our screen. Whilst these proteins have previously been studied in neuronal cardiovascular systems [41], their roles in macrophages have not previously been implicated. However, the importance of intracellular potassium concentration in the activation of NAPL3 inflammasomes have been recently reported [42], thus providing a potential mechanistic explanation for the action of Kcnk channels in the control of *cxcl2* expression.

Interactions between cells and the extracellular matrix have been intensely studied and the importance of this in the control of inflammatory processes is widely recognised. Adhesion molecules, which are critical for the homing of inflammatory cells [43], as well as in their activation, are modified by several groups of enzymes, including sulfotransferases. These proteins have been proposed as therapeutic targets in inflammation [44]. A recent study have demonstrated a distinct expression profile for glyco- and sulfotransferases in monocytes and macrophages, suggesting that specific members of this enzyme superfamily may play a distinct role in the function of monocytes and/or macrophages [45]. Identification of UST, a poorly characterised sulfotransferase fits this general theme whilst also highlighting the power of functional screens to identify novel and specific members of particular gene families.

High throughput approaches are increasingly being used to discover genes involved in the control of mammalian innate immunity. Such strategies include human genome wide association studies (GWAS) of large cohorts with an inflammatory disease, candidate based genetic validation studies, biochemical complementation strategies [1], [4], studies of protein complexes [5], RNAi approaches [7], [46], or overexpression based screens identifying physiologically relevant signalling intermediates [18], [23], [26], [46]–[49]. These approaches complement each other, with one approach identifying candidates whose physiological role is further defined and validated by multiple other approaches. The increase in systems biological resources, together with reagents for loss of function screens including banks of mutant mice (e.g. the KOMP consortium), will aid the rapid investigation of mechanisms of the candidate macrophage inflammatory components discovered here.

## Materials and Methods

### Library construction

cDNA from murine (Balb/c strain) brain and embryo was purchased from Invitrogen (Carlsbad, CA). Splenocytes were isolated from healthy 4–6 week old Balb/c mice. Four cultures of 10^9^ splenocytes were established in RPMI 1640 containing 10% fetal bovine serum. These were stimulated with either 10 µg/ml LPS (E. coli O55:B5 derived, Sigma) or with 1 µg/ml Concanvalin A (Sigma) for 4 or 12 hours. RNA was extracted using Qiagen RNEasy kits and the four samples pooled. Library construction was subcontracted to Invitrogen (Carlsbad, CA). Reverse transcription was performed using a Not I-poly T primer. After second strand synthesis, cDNA was blunted, digested with Not I, and ligated into the pCMV-SPORT6 mammalian expression vector (Invitrogen), which was opened with EcoR V and Not I. Sequencing was done by Agencourt Biosciences (now part of Beckman Coulter) using ABI 3700 machines. The SP6 primer (which lies between the CMV promoter and 5′ end of the polylinker) was used for the 5′ end sequencing and the T7 (reverse) primer was used for the 3′ end.

### Bioinformatic processing

Base calls were performed with Phred [50], trace and vector clipping with Lucy [51], and low complexity regions detected and masked using RepeatMasker (http://www.repeatmasker.org). Traces were mapped to the mouse mm36 NCBI reference sequence (April 2006), chromosome by chromosome, using Blat [52]; all mouse reference sequences and experimental gene models were downloaded 1 February 2007 from NCBI. The position and strand to which the most 5′ portion of each read matched the mouse genome was determined, and 5′ ends were clustered using the QTClust [53] algorithm with radius 250, as implemented in the R flexclust package (see File S3). Clones from each cluster were selected based on the alignment pattern to the genome, such that different isoforms would be represented in the library.

### Plasmids

#### Expression vectors

A range of open reading frames encoding known components of pro-inflammatory signalling systems, without untranslated regions, were inserted into a version of pCMV-SPORT6 mammalian expression vector, whose polylinker was modified by linker ligation. Clones were obtained either by PCR from murine cDNA libraries, or by subcloning from vectors purchased from Invivogen.

#### Reporter vectors

Reporter vectors used include pGL4.*cxcl2* and pGL4.*ifnb*, which contain the mouse *cxcl2* and *ifnb* promoters in pGL4 (Promega) and have been previously described [27]. pGL4.lcn2 contains a −34 to +1404 fragment from murine *lcn2* promoter, subcloned from a reporter vector kindly provided by Dr S Gaffen [54]. As an internal control, pGL4.EF1.rLuc was used; this was constructed by PCR amplification of the human EF1 promoter from pEF-BOS [55] and insertion of the promoter into pGL4 at the Nhe I site.

All vectors were confirmed by sequencing.

#### Cell lines and transient transfections

Raw 264.7 cells were purchased from ATCC (Cat. No: TIB-71) and maintained according to the suppliers' recommendations. All transfections described in this study were performed in 96 well plates. For the cDNA screen, DNA was prepared using the Wizard 96 SV (Promega) system and jetPEI (Polyplus) based transfection was used, as recommended by the manufacturer (250 ng/well plasmid DNA (including 125 ng reporter DNA and 25 ng cDNA expression plasmid/well) and 0.5 µl/well jetPEI). For the “secondary activity screen” all plasmids were purified with EndoFree maxiprep kits (Qiagen). 60 ng of empty pCS6 plasmid, or insert containing plasmid, was mixed with 115 ng of reporter plasmid (either pGL4, pGL4.*cxcl2*, pGL4.*ifn*b, or pGL4.lcn2) and 5 ng of control plasmid pGL4.EF1.rLuc. Transfections were performed with Superfect (Qiagen) 2.5 µl/well, according to the manufacturer's protocol. After transfection, cells were incubated in 100 µl of complete medium (DMEM+10% heat inactivated fetal bovine serum). In some cases, 16 hours after transfection, agonists (typically LPS, E. coli O55:B5, Sigma) were added in 5 µl of PBS to the medium. Cells were lysed 24 hours after stimulation and luciferase assays performed with Dual Luciferase Assay Kits (Promega).

Subsequent transfections for validation and characterisation of the hits were carried out under similar conditions.

### Statistical analysis and identification of hits in the high throughput screen

Gene expression data were normalised, that is expressed as *Photinus* luciferase activity (*cxcl2*) relative to the constitutive *Renilla* luciferase activity (EF1) within each well. Preliminary data analysis suggested that the data were approximately log-normally distributed, so general linear modelling was used to estimate effects of various conditions (including transfection) on log-transformed normalised luciferase activity using Stata 11 and R 2.11 software.

### Effect of inhibitory molecules on hit-induced activation

The DN molecules, used in this study have been described previously [30]–[35]. Briefly, DN-TIRAP encoded a mutant where proline-125 was substituted with histidine. DN-MyD88 expressed a mutant version of MyD88 encoding the death domain (aa 1–151). DN- TRIF encoded only for the TIR domain (aa387–566). Furthermore, it contained a proline to histidine mutation at aa 434 that is essential for activation of the TLR-mediated signalling. DN-TRAM expressed a cysteine to histidine mutant at aa 117. IRAK1-DN expressed only the N-terminal domain of the protein containing the death domain (aa 1–211). TRAF6-DN (aa 289–522) expressed a mutant lacking the N-terminal domain of the protein. DN-Ras encoded a mutant protein where serine 17 was changed to aspargine.

30 ng of “hit” expression plasmid, 30 ng of DN protein expression plasmid, 115 ng of reporter plasmid (pGL4.*cxcl2*) and 5 ng of control plasmid pGL4.EF1.rLuc were co-transfected into Raw 264.7 cells, using Superfect (Qiagen), as described above. Three independent experiments were carried out, all with similar results. Log_2_ luciferase activity was modeled using the glm function in the R software package as a function of inhibitor/activator combination and the activator (since some activators produce more stimulus than others) and experiment (as absolute luciferase levels varied somewhat from experiment to experiment). Significance of change was assessed using contrasts generated by the model.

### Effect of siRNA molecules on LPS and CL075-induced cytokine activation

Raw 264.7 cells were transfected with the *cxcl2*-pLuc and EF1-rLuc reporters, as described above, as well as with 10 pmol siRNA (ON-TARGET *plus* SMART pool, Dharmacon) aginst selected hits that are endogenously expressed in these cells. The impact of siRNA knockdown on LPS (20 ng/ml) or CL075 (2 µg/ml) [56] induced *cxcl2* reporter activation was measured after 6 hrs stimulation. White bars: vehicle, grey bars: agonist added. Data was analysed by two-way ANOVA modelling effect of agonist (LPS or CL075) and siRNA (four categories). Significance was determined from contrast estimates from the model.

## Supporting Information

File S1 **Forward Sequences of cDNA library.** The sequences of forward reads from the cDNA library are shown. Sequences are clipped based on Phred quality scores.
(CSV)

File S2 **Reverse Reads from cDNA library.** The sequences of reverse reads from the cDNA library are shown. Sequences are clipped based on Phred quality scores. No sequence is shown where reverse sequencing did not pass quality control criteria.
(CSV)

File S3 **Mapping of selected clones to the mouse genome.** Sequences of reads were mapped to the mouse mm36 reference genome using Blat. The positions of the 5′ end of each read were clustered to within 250-base pair groups. Positions of the 13,909 clusters generated are shown, mapped to the mm36 mouse genome.
(GFF)

File S4 **Sequences of clones from MGC collections.** Stated sequences of the clones from the MGC collection used to supplement the library are shown.
(CSV)

File S5 **Annotated list of library clones and contents.** Identities of clones obtained from the two-stage library construction process are shown.
(CSV)
